# Association Between Oral Lichen Planus and Non‐Oral Cancers: A Multicentre Case–Control SIPMO Study

**DOI:** 10.1111/odi.70152

**Published:** 2025-11-24

**Authors:** Gioele Gioco, Anna Schiavelli, Alessandra Majorana, Elena Bardellini, Silvio Abati, Andrea Sardella, Francesco Spadari, Matteo Pellegrini, Dorina Lauritano, Raffaele Borgia, Monica Pentenero, Samuele Sutera, Paolo Giacomo Arduino, Alessio Gambino, Roberto Broccoletti, Matteo Biasotto, Giulia Ottaviani, Lorenzo Azzi, Alessandro D'Aiuto, Davide Bartolomeo Gissi, Andrea Gabusi, Andrea Santarelli, Marco Mascitti, Paolo Vescovi, Gloria Bortolotti, Maddalena Manfredi, Marco Meleti, Grazia Zino, Umberto Romeo, Gianluca Tenore, Gianfranco Favia, Luisa Limongelli, Massimo Petruzzi, Francesca Dimilito, Lorenzo Lo Muzio, Maria Eleonora Bizzoca, Giuseppina Campisi, Vera Panzarella, Stefania Leuci, Rosario Serpico, Alberta Lucchese, Giuseppe Colella, Giacomo Oteri, Antonia Marcianò, Gaetano Isola, Alessandro Polizzi, Amerigo Giudice, Elena Calabria, Umberto Mariani, Daniela Adamo, Federica Canfora, Filippo Graziani, Marco Nisi, Michele Giuliani, Luisa Ritrovato, Federica De Micheli, Cosimo Rupe, Francesco Scilla, Romeo Patini, Massimo Cordaro, Michele Davide Mignogna, Carlo Lajolo, SIPMO (Italian Society of Oral Pathology and Medicine)

**Affiliations:** ^1^ Head and Neck Department Fondazione Policlinico Universitario A. Gemelli IRCCS Università Cattolica del Sacro Cuore Rome Italy; ^2^ Department of Medical and Surgical Specialities, Radiological Sciences and Public Health, School of Pediatric Dentistry University of Brescia Brescia Italy; ^3^ Clinical Unit of Oral Medicine and Pathology, Dental School IRCCS San Raffaele Hospital and University Vita‐Salute San Raffaele Milan Italy; ^4^ Dipartimento di Scienze Biomediche, Chirurgiche e Odontoiatriche Università Degli Studi di Milano Milano Italy; ^5^ Department of Biomedical, Surgical and Dental Sciences, Maxillo‐Facial and Dental Unit, Fondazione IRCCS ca' Granda Ospedale Maggiore Policlinico University of Milan Milan Italy; ^6^ Department of Translational Medicine and for Romagna University of Ferrara Ferrara Italy; ^7^ Oral Medicine and Oral Oncology Unit, Department of Oncology University of Turin Turin Italy; ^8^ Department of Surgical Sciences Cir‐Dental School, University of Turin Turin Italy; ^9^ Department of Medicine, Surgery and Health Sciences University of Trieste Trieste Italy; ^10^ Department of Medicine and Technological Innovation University of Insubria Varese Italy; ^11^ Unit of Oral Medicine and Pathology, Dentistry and Oral Diseases Complex Unit ASST Sette Laghi Hospital Varese Italy; ^12^ Department of Biomedical and Neuromotor Sciences, Section of Oral Sciences University of Bologna Bologna Italy; ^13^ Department of Clinical Specialistic and Dental Sciences Marche Polytechnic University Ancona Italy; ^14^ Department of Medicine and Surgery, University Center of Dentistry University of Parma Parma Italy; ^15^ Division of Dermatology Idi‐IRCCS‐FLMM Rome Italy; ^16^ Department of Oral and Maxillo‐Facial Sciences University of Rome “Sapienza” Rome Italy; ^17^ Department of Interdisciplinary Medicine University of Bari “Aldo Moro” Bari Italy; ^18^ Department of Clinical and Experimental Medicine University of Foggia Foggia Italy; ^19^ Department of Precision Medicine in Medical, Surgical and Critical Care (Me.Pre.C.C.) University of Palermo Palermo Italy; ^20^ Unit of Oral Medicine and Dentistry for Fragile Patients, Department of Rehabilitation, Fragility, and Continuity of Care A.O.U.P “P. Giaccone” of Palermo Palermo Italy; ^21^ Department of Neuroscience, Reproductive Sciences and Dentistry University of Naples Federico II Naples Italy; ^22^ Multidisciplinary Department of Medical, Surgical and Dental Specialties University of Campania Luigi Vanvitelli Naples Italy; ^23^ Department of Biomedical and Dental Sciences and Morphofunctional Imaging University of Messina Messina Italy; ^24^ School of Dentistry, Department of General Surgery and Surgical‐Medical Specialties University of Catania Catania Italy; ^25^ School of Dentistry, Department of Health Sciences Magna Graecia University of Catanzaro Catanzaro Italy; ^26^ Department of Dentistry ASST Papa Giovanni XXIII Bergamo Italy; ^27^ Department of Life Science, Health, and Health Professions Link Campus University Rome Italy; ^28^ Department of Surgical Pathology, Medicine, Molecular and Critical Area University of Pisa Pisa Italy; ^29^ Dentistry ‐ IRCCS “Casa Sollievo Della Sofferenza” San Giovanni Rotondo Foggia Italy; ^30^ ASST Cremona, “Ospedale Maggiore di Cremona” Cremona Italy; ^31^ ASST Crema. Ospedale Maggiore, ASST Crema Crema Italy

**Keywords:** comorbidity, extra‐oral cancer, neoplasm, oral lichen planus, SIPMO

## Abstract

**Objectives:**

This multicentre case–control study aimed to investigate the potential association between oral lichen planus (OLP) and extraoral cancers. The secondary objective included the identification of risk factors for this association.

**Methods:**

The study was conducted between January 2023 and June 2024 and included 21 Italian Oral Medicine centres affiliated with the Italian Society of Oral Medicine (SIPMO). The study protocol was approved by the Ethics Committee of Università Cattolica del Sacro Cuore (Prot. ID 4073) and registered at clinicaltrials.gov (ID NCT06449248).

**Results:**

In total, 1650 participants were enrolled (550 OLP and 1100 non‐OLP patients) matched for age and sex. OLP patients showed a significantly higher prevalence of extra‐oral cancers (19.8%) compared to controls (12.4%) with a 1.79 OR (1.34–2.39, *p* < 0.05). Moreover, erosive OLP (*n* = 32/120, 26.7%, *p* < 0.01) and plaque‐like variants (*n* = 27/114, 23.7%, *p* < 0.04) seem to have a slightly increased risk of extra‐oral cancer.

**Conclusions:**

Patients with OLP had a higher risk of developing extraoral cancer; moreover, erosive and plaque‐like forms could be considered at higher risk. Potential pathogenic mechanisms involve an interplay between chronic inflammation, immune dysregulation and carcinogenesis. Therefore, the directionality of such association remains unclear, underscoring the need for prospective studies to clarify causality and temporal dynamics.

## 1 Introduction

Lichen planus (LP) is a chronic inflammatory mucocutaneous disease of unknown aetiology (Nogueira et al. 2015) with different clinical manifestations. These lesions could affect the entire oral mucosa; thus, the term oral lichen planus (OLP) was adopted to identify individuals with oral manifestations alone. The main clinical features of OLP are bilateral, often symmetrical, white lesions (Wickham striae), with a reported prevalence ranging from 0.5% to 2.2% in the general population, and a predilection for females between the third and sixth decades of life (Bardellini et al. 2013; Kövesi and Bánóczy 1973; Pigatto et al. 2011; Rajentheran et al. 1999; Roosaar et al. 2006; Thongprasom et al. 2010; Torrente‐Castells et al. 2010). Few other clinical forms include the plaque form, characterised by predominant hyperkeratosis; the atrophic and erosive forms, in which the inflammatory process is predominant; and ultimately, the bullous form.

Although the etiological factors remain unknown, its pathogenesis is well known, and the role of immune dysregulation in the innate immune system has been widely demonstrated (Ismail et al. 2007). Specifically, epithelial cells express an unknown antigen (both endogenous and exogenous) that acts as a non‐self‐antigen with complex activation of the immune system (cytokines and adhesion molecules) against epithelial cells of the basal layer. This dysregulation of the immune system results in an attack mediated mainly by T lymphocytes and is directed against the keratinocytes of the basal layer of the oral mucosa, with consequent apoptosis and increased replication, with hyperkeratosis, acanthosis and increased rete pegs (Roopashree et al. 2010; El‐Howati et al. 2023).

Several other immune‐mediated disorders are associated with an increased risk of cancer in the involved organs (e.g., inflammatory bowel disease and colorectal cancer [Olén et al. 2020], primary sclerosing cholangitis and hepatobiliary cancer [McGee et al. 2019], celiac disease and small‐bowel cancer [Emilsson et al. 2020]). In addition, OLP is associated with an increased risk of malignant transformation (van der Waal 2009a, 2009b), with rates ranging from 1% to 3% (Giuliani et al. 2019; González‐Moles et al. 2019). Therefore, OLP is a potentially malignant disorder (PMD).

Furthermore, recent studies have highlighted an association between immune‐mediated disorders and an increased risk of distant neoplasms from the involved organs. Crohn's is associated with several distant neoplasms, ulcerative colitis with hepatobiliary cancer and rheumatoid arthritis, with a higher risk of lymphoma and lung cancer (Simon et al. 2015; Mercer et al. 2013). These data suggest that some immune‐mediated diseases may be associated with an increased risk of extra‐local cancer (both solid and haematological tumours), supporting a plausible association between autoimmune diseases, immunosuppression and tumour immunosurveillance (Patini et al. 2023).

Based on a preliminary literature analysis, few studies have reported scattered data on cancer prevalence in patients with OLP. These studies do not provide a definite answer: the majority of them encompass both oral and extra‐oral cancer (Rödström et al. 2004; Dave et al. 2021), while others have a limited sample size (Dave et al. 2021). In other studies, the control group was selected using national registers (no matched group) (Rödström et al. 2004). These limitations, as well as the high variability in the reported cancer prevalence, cannot provide conclusive results. A recent study highlighted a possible association between OLP and the risk of developing distant neoplasms (García‐Pola et al. 2024).

Simultaneously, we conducted a multicentre study involving university centres recognised by the Italian Society of Oral Pathology and Medicine (SIPMO) throughout Italy, with the primary aim of evaluating the possible association between OLP and extraoral cancer, both solid and haematological. The secondary aim was to identify any risk factors (e.g., habits, comorbidities and clinical forms of OLP) that underlie this association.

## 2 Methods

### 2.1 Study Design

This retrospective multicentre case–control observational clinical study was conducted from January 2023 to June 2024 and involved 21 oral medicine centres affiliated with the Italian Society of Oral Pathology and Medicine (SIPMO) in Italy. The coordinates of the coordinating and satellite centres are presented in Appendix S1.

The study was approved by the ethics committee of the promotion centre and satellite centres and was conducted in accordance with the principles of the Declaration of Helsinki (Prot. ID 4073; Clinicaltrial.gov NCT06449248).

The study was conducted following the STROBE guidelines for observational studies.

### 2.2 Study Population

Twenty‐five patients affected by OLP and 50 patients in the control group, matched by sex and age (within a range of 5 years), were enrolled in this multicentre study at each centre, and 1650 patients (550 patients affected by OLP—case group; 1100 control group) were enrolled in this case–control study.

The patients included in the case and control groups were selected consecutively at Oral Medicine clinics recognized by the SIPMO.

### 2.3 Inclusion Criteria

Group 1 (case group):

- Both sexes
  ○ Clinical and histological diagnoses of OLP were made according to the WHO diagnostic criteria modified by van der Meij et al. (2003). The clinical diagnosis of OLP was achieved using the following criterion: presence of a bilateral, more or less symmetrical, lace‐like network of slightly raised grey‐white striae. Erosive, atrophic, bullous and plaque‐type lesions are accepted subtypes of OLP. The histopathological criteria were as follows: (1) a well‐defined band‐like zone of cellular infiltration confined to the superficial part of the connective tissue, consisting mainly of lymphocytes; (2) signs of liquefaction degeneration in the basal cell layer; and (3) absence of epithelial dysplasia. A final diagnosis of OLP was made, fulfilling both the clinical and histopathological criteria at each SIPMO centres.

Group 2 (control group):

- Both gender
  ○ Outpatients availing dental services who came for dental checkups (professional hygiene and conservative therapies) in the absence of suspicious lesions of OLP.

### 2.4 Exclusion Criteria

Group 1 (case group):

- Patients presenting with dysplasia or OSCC at diagnosis were excluded to reduce the possible false‐positive cases of OLP‐similar lesions. To this regard, it is essential to note that immunosurveillance can occur beneath dysplastic epithelium or cancer, potentially mimicking the band‐like lympho‐monocytic infiltrate typical of OLP (Stojanov et al. 2024). Conversely, OLP is considered a PMD and may present as a dysplastic epithelium or carcinoma during malignant transformation (Lodi et al. 2005). Due to the lack of consensus regarding the possible presence of dysplasia or OSCC at diagnosis, patients with dysplasia or OSCC at the first diagnosis were excluded from the study.
- Patients with head and neck cancer were excluded as they have a high risk of both synchronous and metachronous second primary cancers (SPC). The increasing risk of SPC is related to various reasons concerning the theory of ‘field cancerization’, including genetic predisposition, carcinogenic cancer treatments and mostly, environmental and lifestyle risk factors shared by the first and second cancers. This theory hypothesises that exposure to carcinogenic factors (e.g., smoking and alcohol) could damage genes in the epithelial layer of the entire area, leading to an increased risk of malignant lesions in distant sites of the head and neck. Therefore, we decided to exclude all HNC, as they share the main risk factors for OSCC.
- Patients with oral lichenoid lesions (OLL) were also excluded according to the recent WHO classification of OPMD (Warnakulasuriya et al. 2021). OLL encompass a group of diseases: atypical OLP and unilateral lichenoid lesions; those in close contact relationship to a dental restoration, often amalgam, referred to as oral lichenoid contact reactions (OLCR); lichenoid drug‐reactions (LDR), oral lesions following intake of food or some substances (e.g., cinnamon); and oral lesions of graft versus host disease (GVHD). Specifically, patients who received a haematopoietic stem cell transplant (HSCT) for blood malignancies and developed oral GVHD with OLP‐like lesions were also excluded.

Group 2 (control group):

- History of OLP diagnosis (clinical and/or histological diagnosis)
- Patients with dysplastic oral lesions, OSCC, head and neck cancer, OLL were excluded for the same reasons as those in the case group.

In order to enhance diagnostic concordance across centres, a calibration meeting was conducted among the principal investigators of the participating SIPMO centres to reach a consensus on diagnostic criteria, particularly for challenging cases (OLP and OLL), based on the recommendations of Warnakulasuriya et al. (2021). Each centre is led by a clinician with at least 10 years of experience in oral medicine.

### 2.5 Outcome

The primary outcome was the association between OLP and the history of extraoral cancer (i.e., previous, simultaneous or subsequent to the diagnosis of OLP), including both solid and haematological neoplasms.

The secondary outcome was to investigate any possible risk factors: clinical forms of OLP, specific oncological pathologies, smoking and alcohol use and/or the presence of other comorbidities (e.g., diabetes, hypertension, chronic liver disease, HPV infection and HCV infection). Another outcome focused on the evaluation of the timing of the onset of OLP and extraoral cancer.

### 2.6 Data Collection

The study was conducted by analysing the data collected from the medical records of patients enrolled in each Oral Medicine centre.

Data on sociodemographic, anamnestic and clinical characteristics were extrapolated and collected using an ad hoc electronic spreadsheet. Specifically, the clinical features of OLP were classified as: reticular form; plaque form; atrophic form; erosive form; bullous form. If present, the history of extraoral cancer was collected as previous, simultaneous or subsequent to the diagnosis of OLP.

In addition, data on oncologic therapy, systemic treatments for other conditions and potential comorbidities were recorded if reported in the medical records and were investigated as potential risk factors for OLP.

### 2.7 Sample Size and Statistical Analysis

The total sample size was calculated from a preliminary analysis of 84 medical records from our centre (42 cases and 42 controls) and an odds ratio (OR) of 2.4 was obtained.

Considering the prevalence of malignant neoplasms in the world population of 6% (Ritchie 2015) assuming a control‐to‐case ratio of 2:1 and setting the power of the study at 95% and an alpha error of 0.05%, it was necessary to enrol 506 cases and 1011 controls for a total of 1517 enrolled patients. Calculations were performed using the EpiInfo StatCalc program (7.2.4.3).

Qualitative variables are described using absolute and percentage frequencies, whereas quantitative variables are summarised as means and standard deviations (SD) for normally distributed data or as medians and percentiles for non‐normally distributed data. The Kolmogorov–Smirnov test was performed to evaluate the normal distribution of the quantitative variables.

Differences between the case and control groups were assessed as follows: Mann–Whitney *U* and Kruskal–Wallis tests were performed to compare continuous variables with non‐parametric distributions. Parametric variables were analysed using ANOVA. Pearson's Chi‐squared test and Fisher's exact test were utilised to compare discontinuous variables between groups.

Variables identified as risk factors in the univariate analysis (*p* < 0.05) were entered into a stepwise multiple logistic regression model, with the presence of OLP as the dependent variable. Odds ratios (ORs) and 95% confidence intervals were determined. Goodness of fit of the multivariate logistic regression model was evaluated by means of the Hosmer–Lemeshow test. Statistical analyses were performed using Statistical Package for Social Sciences software (IBM Corp. Released 2012. IBM SPSS Statistics for Windows, Version 21.0 Armonk, NY: IBM Corp).

## 3 Results

A total of 1650 patients were enrolled in this multicentre case–control study (550 patients with OLP: case group; 1100 controls), with a mean age of 62.00 years (10–99. SD: 12.4): 698 (42.3%) male patients and 952 (57.7%) female patients were enrolled.

The general characteristics of both the case and control groups are reported in Table 1.

**TABLE 1:** Demographic characteristics of the included patients.

Variables	Case group—OLP (*n* = 550)	Control group—non OLP (*n* = 1100)	*p*
Age, years, mean (min–max; SD)			Mann–Whitney—*p* = 0.5
	63.22 (24–95; 12.5)	61.39 (10–99; 12.3)	
Region, *N* (%)			*χ* ^2^ test—*p* = 0.98
Northen Italy	292 (53.1)	582 (52.9)	
Centre Italy	127 (23.1)	255 (23.2)	
Southern Italy	131 (23.8)	263 (23.9)	
Gender, *N* (%)			*χ* ^2^ test—*p* = 0.27
Male	222 (40.4)	476 (43.3)	
Female	328 (59.6)	624 (56.7)	
Smoke, *N* (%)			*χ* ^2^ test—*p* = 0.55
Yes	105 (19.1)	234 (21.3)	
No	445 (80.9)	866 (78.7)	
Job, *N* (%)			*χ* ^2^ test—*p* = 0.57
Unemployed	73 (13.3)	112 (10.2)	
Employed	140 (25.5)	227 (20.6)	
Self‐employed	46 (8.4)	82 (7.5)	
Retired	148 (26.9)	203 (18.5)	
nd	143 (26.0)	476 (43.3)	
Social status, *N* (%)			*χ* ^2^ test—*p* = 0.39
Single	59 (10.7)	105 (9.6)	
Married	278 (50.6)	392 (35.6)	
Divorced	17 (3.1)	35 (3.2)	
Widowed	28 (5.1)	47 (4.3)	
nd	168 (30.6)	521 (47.4)	
Alcohol consumption, *N* (%)			*χ* ^2^ test—***p* < 0.0001**
Yes	501 (91.1)	1057 (96.1)	
No	49 (8.9)	43 (3.9)	

*Note:* Bold value indicates significant.

Regarding the clinical features of patients with OLP, 329 had reticular OLP (59.8%), 114 had plaque forms (20.7%), 92 had atrophic forms (16.7%), 120 had erosive forms (21.8%) and 21 had bullous forms (3.8%).

The main variables, including age, sex, smoking and centre of origin, were distributed homogeneously in the sample with no statistical differences (*p* > 0.05). A comparison of the main variables between the case and control groups is shown in Table 2.

**TABLE 2:** Characteristics of the included sample, according to OLP patients and control group.

Variables	Case group—OLP (*n* = 550)	Control group—non OLP (*n* = 1100)	*p*
Neoplasm, *N* (%)			*χ* ^2^ test—***p* < 0.0001**
Yes	109 (19.8)	136 (12.4)	
No	441 (80.2)	964 (87.6)	
Type of neoplasm *N* (%)			
*Total*	109 patients	136 patients	
Breast cancer	49 (8.9)	73 (6.63)	*χ* ^2^ test—*p* = 0.19
Prostate cancer	6 (1.1)	14 (1.3)	*χ* ^2^ test—*p* = 0.24
Lung cancer	4 (0.73)	1 (0.09)	*χ* ^2^ test—*p* = 017
Stomach cancer	6 (1.1)	5 (0.45)	*χ* ^2^ test—*p* = 0.55
Colon cancer	4 (0.73)	4 (0.36)	*χ* ^2^ test—*p* = 0.51
Blood neoplasm	6 (1.1)	10 (0.91)	*χ* ^2^ test—*p* = 0.61
Skin cancer	4 (0.73)	6 (0.54)	*χ* ^2^ test—*p* = 1
Thyroid cancer	9 (1.64)	5 (0.45)	*χ* ^2^ test—*p* = 0.17
Kidney cancer	2 (0.36)	11 (1)	*χ* ^2^ test—***p* = 0.04**
Ovarian cancer	3 (0.54)	3 (0.27)	*χ* ^2^ test—*p* = 0.53
Uterine cancer	7 (1.27)	—	*χ* ^2^ test—***p* = 0.03**
Bladder cancer	3 (0.54)	1 (0.09)	*χ* ^2^ test—*p* = 0.33
Liver cancer	2 (0.36)	2 (0.18)	*χ* ^2^ test—*p* = 1
Adrenal cancer	2 (0.36)	—	*χ* ^2^ test—*p* = 0.19
Thymic cancer	1 (0.18)	—	*χ* ^2^ test—*p* = 0.38
Brain cancer	1 (0.18)	1 (0.09)	*χ* ^2^ test—*p* = 1
Oncological history *N* (%)			*χ* ^2^ test—*p* = 0.69
Single cancer	96 (17.45)	127 (11.5)	
Multiple cancer	13 (2.36)	9 (0.82)	
Diabetes			*χ* ^2^ test—***p* = 0.03**
No	474 (86.2)	923 (83.91)	
Type 1	10 (1.8)	8 (0.73)	
Type 2	66 (12)	169 (15.36)	
Cardiovascular diseases			*χ* ^2^ test—***p* = 0.05**
No	334 (60.7)	722 (65.6)	
Yes	216 (39.3)	378 (34.4)	
Ischemic	40 (7.3)	72 (6.6)	*χ* ^2^ test—*p* = 0.6
Hypertension	162 (29.5)	285 (26.9)	*χ* ^2^ test—*p* = 0.13
Other	14 (2.6)	21 (1.9)	*χ* ^2^ test—*p* = 0.24
Liver diseases			
No	504 (91.9)	1054 (95.8)	
Yes	46 (8.1)	46 (4.2)	*χ* ^2^ test—***p* = 0.001**
Alcoholic hepatitis	5 (0.9)	22 (2.0)	*χ* ^2^ test—*p* = 0.15
Autoimmune	1 (0.2)	3 (0.3)	*χ* ^2^ test—*p* = 1
HBV	15 (2.7)	10 (0.9)	*χ* ^2^ test—***p* = 0.009**
HCV	23 (4.2)	11 (1.0)	*χ* ^2^ test—***p* < 0.0001**
HAV	2 (0.4)	0 (0.0)	*χ* ^2^ test—*p* = 0.11
Blood diseases			
No	523 (95.1)	1059 (96.3)	
Yes	27 (4.9)	41 (3.7)	*χ* ^2^ test—*p* = 0.07
Anaemia	12 (2.2)	13 (1.2)	*χ* ^2^ test—*p* = 0.14
Blood neoplasm	6 (1.1)	10 (0.9)	*χ* ^2^ test—*p* = 0.11
Haemorrhagic	5 (0.9)	8 (0.7)	*χ* ^2^ test—*p* = 0.77
Coagulative	4 (0.7)	10 (0.9)	*χ* ^2^ test—*p* = 0.79
Renal diseases			
No	535 (97.3)	1076 (97.8)	
Yes	15 (2.7)	24 (2.2)	*χ* ^2^ test—*p* = 0.49
Allergic diseases			
No	523 (95.1)	1045 (95.0)	
Yes	27 (3.9)	55 (5.0)	*χ* ^2^ test—*p* = 1
Bone diseases			
No	468 (85.1)	964 (87.6)	
Yes	82 (14.9)	136 (12.4)	*χ* ^2^ test—*p* = 0.17
Brain diseases			
No	524 (95.3)	1064 (96.7)	
Yes	26 (4.7)	36 (3.3)	*χ* ^2^ test—*p* = 0.17
Thyroid diseases			
No	453 (82.4)	985 (89.6)	
Yes	97 (17.6)	115 (10.4)	*χ* ^2^ test—***p* < 0.0001**
Autoimmune diseases			
No	491 (89.3)	1064 (96.7)	
Yes	59 (10.7)	36 (3.39)	*χ* ^2^ test—***p* < 0.0001**
Other comorbidities			
No	383 (69.6)	844 (76.7)	
Yes	167 (30.4)	256 (23.3)	*χ* ^2^ test—***p* = 0.002**
ACE_Inhibitors			
No	487 (88.6)	1010 (91.8)	
Yes	63 (11.4)	90 (8.2)	*χ* ^2^ test—***p* = 0.04**
Calcium channel blockers			
No	492 (89.5)	1032 (93.8)	
Yes	58 (10.5)	68 (6.2)	*χ* ^2^ test—***p* = 0.002**
Angiotensin II receptor blockers			
No	491 (89.3)	1005 (91.4)	
Yes	59 (10.7)	95 (8.6)	*χ* ^2^ test—*p* = 0.18
Diuretics			
No	519 (94.4)	1055 (95.9)	
Yes	31 (5.6)	45 (4.1)	*χ* ^2^ test—*p* = 0.17
Beta‐blockers			
No	464 (84.4)	967 (87.9)	
Yes	86 (15.6)	133 (12.1)	*χ* ^2^ test—*p* = 0.55
Statins			
No	472 (85.8)	990 (90.0)	
Yes	78 (14.2)	110 (10.0)	*χ* ^2^ test—***p* = 0.01**
Oral hypoglycaemic agents			
No	494 (89.8)	1037 (94.3)	
Yes	56 (10.2)	63 (5.7)	*χ* ^2^ test—***p* = 0.002**
Insulin			
No	535 (97.3)	1054 (95.8)	
Yes	15 (2.7)	46 (4.2)	*χ* ^2^ test—*p* = 0.17
Antiplatelet agents			
No	466 (84.7)	987 (89.7)	
Yes	84 (15.3)	113 (10.3)	*χ* ^2^ test—***p* = 0.004**
Anticoagulants			
No	533 (96.9)	1071 (97.4)	
Yes	17 (3.1)	29 (2.6)	*χ* ^2^ test—*p* = 0.64
Bone antiresorptive drugs			
No	524 (95.3)	1046 (95.1)	
Yes	26 (4.7)	54 (4.9)	*χ* ^2^ test—*p* = 0.9
Levotiroxine			
No	486 (88.4)	1026 (93.3)	
Yes	64 (11.6)	73 (6.7)	*χ* ^2^ test—***p* = 0.001**
Proton pump inhibitors			
No	472 (85.8)	990 (90.0)	
Yes	78 (14.2)	110 (10.0)	*χ* ^2^ test—***p* = 0.01**
Steroids			
No	535 (96.7)	1075 (97.7)	
Yes	15 (3.3)	25 (2.3)	*χ* ^2^ test—*p* = 0.61
Sulfasalazine			
No	544 (98.9)	0 (99.4)	
Yes	6 (1.1)	6 (0.6)	*χ* ^2^ test—***p* = 0.001**
Azathioprine			
No	547 (99.5)	1099 (99.9)	
Yes	3 (0.5)	1 (0.1)	*χ* ^2^ test—*p* = 0.11
Other drugs			
No	350 (63.6)	826 (75.1)	
Yes	200 (36.4)	274 (25.9)	*χ* ^2^ test—***p* < 0.0001**
Radiotherapy			
No	28 (25.7)	46 (33.8)	
Yes	48 (44.0)	33 (24.3)	
nd	33 (30.3)	57 (41.9)	*χ* ^2^ test—*p* = 0.11
Oncological surgery			
No	68 (62.4)	66 (48.6)	
Yes	8 (7.3)	13 (9.6)	
nd	33 (30.3)	57 (41.9)	*χ* ^2^ test—*p* = 0.61
Chemotherapy			
No	31 (28.4)	34 (25.0)	
Yes	45 (41.3)	45 (33.1)	
nd	33 (30.3)	57 (41.9)	*χ* ^2^ test—*p* = 0.53

*Note:* Bold value indicates significant.

Overall, 109 of 550 patients with OLP (19.8%) were affected by extraoral cancer compared to 136 of 1100 patients in the control group (12.4%, *p* < 0.05).

At univariate analysis, several covariates were associated with OLP: lifestyle factors (i.e., alcohol consumption *p* < 0.0001); comorbidities (i.e., diabetes *p* = 0.03, cardiovascular diseases *p* = 0.05, liver diseases *p* = 0.001, HBV infection *p* = 0.009, HCV infection *p* < 0.0001, thyroid disorders *p* < 0.0001, autoimmune diseases *p* < 0.0001 and other comorbidities *p* = 0.002); systemic pharmacological treatments (i.e., ACE inhibitors *p* = 0.04, calcium channel blockers *p* = 0.002, statins *p* = 0.01, oral hypoglycaemic agents *p* = 0.002, antiplatelet drugs *p* = 0.004, levothyroxine *p* = 0.001, proton pump inhibitors *p* = 0.01, sulfasalazine *p* = 0.001) and neoplasms (i.e., uterine cancer *p* = 0.03 and kidney cancer *p* = 0.04). In particular, a higher prevalence of uterine cancer was found in patients with OLP (seven cases vs. zero), while kidney cancer was more frequent among non‐OLP controls (11 cases vs. two). The results of univariate analysis are reported in Tables 1 and 2.

No association was found between oncologic treatments (radiotherapy, surgery and chemotherapy) and OLP (*p* > 0.05).

At the multivariate analysis, the extra‐oral cancer was associated with OLP with an OR = 1.79 (1.34–2.39, *p* < 0.05), thus representing an independent risk factor. Additionally, at logistic regression other independent risk factors were identified: alcohol consumption, liver diseases, HBV infection, HCV infection, thyroid disorders, autoimmune diseases and the use of hypoglycaemic agents. No specific cancer site remained significantly associated with OLP. The results of the logistic regression are presented in Table 3. The Hosmer–Lemeshow test resulted not to be statistically significant (*p* = 0.565), thus suggesting a good adaptation of the model to available data.

**TABLE 3:** Results of multivariable logistic regression with adjusted odds ratios (ORs) for oral lichen planus.

OLP			
Predictors	OR	95% CI	*p*
Oncological history			
No	1.0 (Ref)		
Yes	1.79	1.34–2.39	**< 0.0001**
Kidney cancer			
No	1.0 (Ref)		
Yes	0.21	0.04–1.01	0.06
Uterine cancer			
No	1.0 (Ref)		
Yes	1.06	1.01–1.22	0.99
Diabetes			
No	1.0 (Ref)		
Type 1	1.05	0.37–2.98	0.92
Type 2	0.42	0.28–0.64	**< 0.0001**
Liver diseases			
No	1.0 (Ref)		
Yes	1.93	1.25–3.00	**0.003**
HBV infection			
No	1.0 (Ref)		
Yes	2.79	1.19–6.47	**0.017**
HCV infection			
No	1.0 (Ref)		
Yes	4.56	2.17–9.60	**< 0.0001**
Thyroid diseases			
No	1.0 (Ref)		
Yes	1.49	1.10–2.04	**0.011**
Levotiroxine			
No	1.0 (Ref)		
Yes	0.91	0.55–1.50	0.70
Cardiovascular diseases			
No	1.0 (Ref)		
Yes	0.98	0.76–1.26	0.86
ACE inhibitors			
No	1.0 (Ref)		
Yes	1.28	0.87–1.91	0.21
Calcium channel blockers			
No	1.0 (Ref)		
Yes	1.41	0.93–2.14	0.11
Other systemic diseases			
No	1.0 (Ref)		
Yes	1.09	0.84–1.41	0.51
Autoimmune diseases			
No	1.0 (Ref)		
Yes	3.12	1.99–4.89	**0.0001**
Statins			
No	1.0 (Ref)		
Yes	1.15	0.79–1.67	0.46
Oral hypoglicaemic agents			
No	1.0 (Ref)		
Yes	3.38	2.10–5.75	**0.0001**
Antiplatelet agents			
No	1.0 (Ref)		
Yes	1.1	0.76–1.6	0.59
Gastroprotective agents			
No	1.0 (Ref)		
Yes	0.99	0.69–1.41	0.96
Sulfasalazine			
No	1.0 (Ref)		
Yes	1.64	1.01–1.92	0.99
Other drugs			
No	1.0 (Ref)		
Yes	1.14	0.89–1.48	0.29
Alcohol consumption			
No	1.0 (Ref)		
Yes	2.10	1.35–3.26	**0.001**

*Note:* Bold value indicates significant.

Stratified analysis was conducted among the clinical forms of OLP: specifically, erosive OLP (*n* = 32/120, 26.7%, *p* < 0.01) and plaque‐like variants (*n* = 27/114, 23.7%, *p* < 0.04) seem to have a slightly increased risk of extra‐oral cancer, representing a hypothesis‐generating observation (Table 4).

**TABLE 4:** Stratified analysis was conducted among the clinical forms.

Variables	Cancer patients (*n* = 245)	No cancer patients (*n* = 1405)	*p*
OLP patients			*p* < 0.05
Yes	109 (44.5)	441 (31.4)	
No	136 (55.5)	964 (68.7)	
Clinical features, *N* (%)			
Reticular 329	68 (62.4)	261 (59.2)	*p* = 0.07
Plaque 114	27 (24.8)	87 (19.7)	*p* = 0.04
Atrophic 92	18 (16.5)	74 (16.8)	*p* = 0.12
Erosive 120	32 (29.4)	88 (20.0)	*p* = 0.01
Bollous 21	2 (1.8)	19 (4.3)	*p* = 0.06

Further analysis was performed on the time lapse between OLP and extraoral cancer. The OLP diagnosis seemed to occur after the oncological diagnosis: out of 109 OLP patients affected by extraoral cancer, 65 patients (59.6%) received the OLP diagnosis after the oncological diagnosis with a mean time interval of 109.47 months (12–492; SD: 101.72), whereas 10 patients (9.2%) received the OLP diagnosis before the oncological diagnosis with a mean time interval of 51.67 months (18–80; SD: 31.34); only 2 patients received a simultaneous diagnosis (1.8%, *p* < 0.001). However, it was not possible to establish a temporal connection between the two diagnoses in 32 patients (29.4%) (Table 5).

**TABLE 5:** The time lapse between OLP and extraoral cancer.

Time to diagnosis of OLP, *N* (%)	*N* (%)	Mean time (max–min, SD)
OLP patients diagnosis*		
After cancer	65 (59.6%)	109.47 months (12–492; 101.72)
Before cancer	10 (9.2%)	51.67 months (18–80; SD: 31.34)
nd	32 (29.4%)	—
Simultaneously	2 (1.8%)	0

* *p* < 0.001.

## 4 Discussion

This multicentre case–control study highlighted that OLP is associated with an increased risk of extraoral cancer in a group of outpatients attending university SIPMO centres throughout Italy with a slightly greater predilection for erosive and plaque clinical features.

Based on a preliminary literature analysis, few studies have reported scattered data on cancer prevalence in patients with OLP. The first study was conducted by Rödström et al. (2004) in a group of 1028 OLP patients during a mean follow‐up of 6.8 years (range: 0.25–16.0 years; SD = 4.9) (Rödström et al. 2004). The study reported an incidence of 55 cases of cancer in the enrolled patients with a standardised incidence ratio of 1.1 (95% CI: 0.9–1.5, *p* > 0.05). Nevertheless, the inclusion of both oral and extra‐oral cancers and the absence of a matched control group represent the main limitations of this study. Another critical issue is related to the inclusion criteria. Study enrolment was conducted from 1978 to 1993, before the WHO criteria were modified by van der Meij and van der Waals. Twenty years later, Dave et al. (2021) investigated the association between OLP and various systemic conditions, including cancer (Dave et al. 2021), using a case–control study. The results corroborated that patients with any type of cancer were approximately three times more likely to be affected by OLP (OR: 3.4, 95% CI: 1.4–8.4). Nevertheless, the small sample size, unmatched control group and inclusion of both oral and extraoral cancers in cancer prevalence cannot provide conclusive results. Together with our study, García‐Pola et al. (2024) reported an association between the history of cancer and OLP with an OR = 2.21 (*p* < 0.05) (García‐Pola et al. 2024). Nevertheless, the single‐centre retrospective study design and the less restrictive inclusion criteria led to some differences in the results. To date, our study is the first multicentre study with a large cohort of patients that report a statistically significant association between OLP and extra‐oral cancer with an OR = 1.79 (1.34–2.39, *p* < 0.005), suggesting an intimate relationship between chronic inflammatory status, immune system and carcinogenesis.

Further consideration should be given to the main risk factors for OLP (e.g., age, sex and infection). This case–control study was matched for age and sex; therefore, no differences were observed between the two cohorts. Moreover, tobacco use was not identified as a risk factor for OLP due to the homogeneous distribution of smokers between the two cohorts (19% in the case group vs. 21% in the control group).

In the multivariate logistic regression analysis, several variables emerged as independent risk factors for OLP. Alcohol consumption increases the risk of OLP (OR = 2.10; 1.35–3.26, *p* < 0.005): chronic ethanol and its metabolites exposure can alter the epithelial barrier, increase mucosal permeability and facilitate the penetration of antigens or irritants, thereby promoting local inflammatory responses (Hoes et al. 2021). Moreover, ethanol can modulate both innate and adaptive immune responses, including alterations in T‐cell function and the Th1/Th2 balance, which may play a role in the pathogenesis of immune‐mediated diseases such as OLP (Tharmalingam et al. 2024). In addition, alcohol consumption can interfere with normal nutrition in multiple mechanisms, leading to deficiencies in micronutrients. The consequent malnutrition impairs the integrity of the mucosal barrier and the proper functioning of the immune system increasing the risk of both OLP and cancer (Gholizadeh and Sheykhbahaei 2021; Patini et al. 2024).

Liver diseases, including those related to HBV and HCV infection, were also significantly associated, particularly HCV infection likely through mechanisms involving chronic inflammation and immune dysregulation. The role of HCV infection in the pathogenesis of OLP remains unclear, probably because of the specific direct immune response to HCV‐infected epithelial cells (Alaizari et al. 2016). HCV infection is a well‐known epidemic in Mediterranean countries and Japan, whereas in Anglo‐Saxon countries, it is much less common (Giuliani et al. 2007). Our study reported HCV infection as a major OLP independent risk factor with an OR of 4.57 (95% CI: 2.17–9.60, *p* < 0.05) and HBV infection with an OR of 2.79 (95% CI: 1.2–6.47, *p* < 0.05); nevertheless, it is possible that, due to the high HCV infection rate in Italy, this association is only due to an epidemiologic association as already discussed by Giuliani et al. (2007). However, it was not possible to determine the temporal relationship between HCV infection and the development of oral lesions.

Regarding systemic diseases, the autoimmune diseases were also associated with OLP, supporting the hypothesis of an immune‐mediated pathogenesis of OLP. These results are corroborated by a previous systematic review that reported the existence of a comorbidity between autoimmune diseases and OLP (De Porras‐Carrique et al. 2023).

Considering other systemic diseases, Type 2 diabetes appeared to be inversely associated with OLP, a finding that contrasts with prior reports and may be due to selection bias, or the potential protective effect of certain antidiabetic medications (Adamo et al. 2023). In this regard, it should be noted that while Type 1 diabetes is an autoimmune disease, making its association with OLP more easily understandable, Type 2 diabetes is a metabolic syndrome associated with chronic low‐grade inflammation but also with impaired immune function (Adamo et al. 2021). This reduced immune reactivity of both innate and adaptive immune responses could, paradoxically, decrease the likelihood of mounting the type of T cell–mediated inflammatory response involved in the pathogenesis of OLP, thereby appearing as a protective factor in our model (Berbudi et al. 2020). However, this hypothesis remains speculative and warrants confirmation in future prospective studies.

Among systemic therapies, oral hypoglycaemic agents were identified as risk factors for OLP even in the multivariate regression analysis. This finding could be partially interpreted as a possible lichenoid drug reaction, although robust evidence supporting this association is lacking. Moreover, it was not possible to determine the temporal relationship between drug intake and the development of oral lesions. Data regarding DM and its therapy, both for Type 1 and Type 2, should be better investigated in a specific study.

Oncological treatment both chemotherapy and radiotherapy was not a risk factor for OLP.

According to the type of cancer, the study conducted by García‐Pola et al. (2024) reported statistically significant differences between the OLP patients' group and the control group for breast cancer with OR = 3.71 (CI = 1.03–13.38; *p* < 0.05). Moreover, cervical and colon cancers were more prevalent in patients with OLP, although the differences were not statistically significant (*p* > 0.05). Although our study reported a higher prevalence of uterine cancer in patients with OLP (seven cases vs. 0), the multivariate analysis was not statistically significant, probably due to the limited number of uterine cancers. This slight prevalence could be partly influenced by the vulvar involvement of lichen planus (Janovska et al. 2023). To date, vulvar lichen sclerosus seems to represent a risk factor for developing vulvar cancer or its precursors (Vieira‐Baptista et al. 2022). Nevertheless, due to the retrospective nature of the study, it was not possible to retrieve the genital involvement of patients affected by OLP. Therefore, further studies should be conducted to investigate in a specific manner the association between uterine cancer and OLP patients.

Although its pathogenesis is unknown, several rationales may underlie the association between OLP and extraoral cancer. First, a plausible rationale could be autoimmunity triggered by the neoplasia itself. The neoplasm interacts with the host immune system and leads to the development of chronic immune‐mediated inflammatory diseases. Neoplastic cells express some molecules called tumour antigens, stimulating the immune response (Vigneron 2015). Tumour antigens are classified into tumour‐associated antigens (TAA) and tumour‐specific antigens (TSA) (Coulie et al. 2014). Tumour‐associated antigens are also expressed in healthy tissues, but at lower concentrations. On the other hand, tumour‐specific antigens are molecules expressed exclusively by neoplastic cells and are molecules contained in a specific cellular compartment (i.e., membrane, cytoplasm, nucleus) and/or released into the bloodstream. Recently, a novel group of tumour‐specific antigens was defined as neoantigens, which represent new antigens generated by tumour cells following various tumour‐specific alterations, such as genomic mutations, dysregulated RNA splicing and altered post‐translational modifications of proteins (Tokita et al. 2024). Neoantigens are recognised as non‐self antigens and trigger an immune response, avoiding the classic phenomena of central and peripheral immune tolerance (Sakowska et al. 2022). Furthermore, the stromal environment, characterised by necrosis and inflammatory molecules, can lead to the anomalous release of autoantigens, activating the immune response in an uncontrolled manner (Nanda and Sercarz 1995). Therefore, tumour antigens (tumour‐specific antigens, neoantigens and stromal autoantigens) mimic proteins expressed in healthy tissues and induce the immune system to lose self‐tolerance. This cross‐reaction with tumour antigens is well known in other conditions known as paraneoplastic autoimmune disorders (PADs) which represent a heterogeneous group of autoimmune diseases with no apparent connection to neoplasia (Geng et al. 2020). The primary known PADs include paraneoplastic pemphigus, systemic sclerosis, thymoma‐associated autoimmunity, haemolytic anaemia and pure red blood cell aplasia (Weksler and Lu 2014). Thus, LPO may be a sign of distant neoplasia.

A second plausible rationale is the immune dysregulation of patients with OLP. Among the main pathogenic mechanisms of OLP, there are some specific immunoregulatory mechanisms, including T helper 17 lymphocytes, which promote the inflammatory state, and the dysfunction of Treg lymphocytes, which are involved in the immune tolerance mechanism (He et al. 2022). Therefore, impairment of the immune system can result in inadequate immunosurveillance, interfering with the mechanisms of identification and destruction of neoplastic cells at an early stage (Sakowska et al. 2022). Furthermore, prolonged therapies based on immunosuppressive drugs adopted by patients could contribute to immune evasion by neoplastic cells (Patini et al. 2023).

A third plausible explanation could be related to the systemic inflammatory status of patients with OLP (i.e., chronic inflammation). Although the risk of local carcinogenesis is now well established, OLP is a PMD with a malignant transformation rate varying from 1% to 3% (Giuliani et al. 2019; González‐Moles et al. 2019), and the chronic inflammatory status supported by inflammatory cells and mediators (i.e., cytokines, chemokines and free radicals) could increase the risk of distant carcinogenesis (He et al. 2022). For instance, an increased risk of neoplasia at distant sites has been reported in other chronic immune‐mediated inflammatory diseases (e.g., ulcerative colitis with prostate cancer, autoimmune hepatitis with oesophageal and tongue cancer, and idiopathic thrombocytopenic purpura with liver cancer) (Emilsson et al. 2020; Simon et al. 2015).

As highlighted in previous reports, chronic systemic inflammation can promote both carcinogenesis and the persistence of immune‐mediated disorders such as OLP. T‐cell dysregulation, particularly involving cytotoxic CD8^+^ lymphocytes, is a hallmark of OLP and may also contribute to tumour immunoediting (Vale et al. 2023), Furthermore, activation of the PI3K/Akt/mTOR pathway is implicated in cell proliferation, survival and metabolic reprogramming and has been observed in both malignant tissues and in the inflamed oral mucosa of OLP patients (Sonis and Amaral Mendes 2016). Together, these mechanisms provide a biologically plausible link between systemic carcinogenesis and OLP, suggesting that the two conditions may share overlapping pathogenic pathways rather than representing entirely distinct disease processes.

Although case–control studies were not designed to investigate the temporal correlation between the two events, in relation to the time interval between OLP diagnosis and oncological diagnosis, we found that most OLP cases were diagnosed after the oncological diagnosis (*n* = 65) with a mean time interval of 109.47 months (12–492; SD: 101.72), and only 10 cases of neoplasia after the diagnosis of OLP with a mean time interval of 51.67 months (18–80; SD: 31.34) (Table 5).

In our cohort, cancer diagnosis generally preceded the diagnosis of OLP by several years. This temporal sequence weakens the argument that OLP could act as a pro‐oncogenic condition. Instead, it supports alternative explanations, such as paraneoplastic phenomena or immune surveillance failure. In this context, autoimmunity may be triggered by a cross‐reaction to unknown tumour antigens, maintaining a chronic immune‐mediated inflammatory state. However, the direction of this association remains unclear, highlighting the need for prospective studies to clarify causality and temporal dynamics.

### 4.1 Strengths and Limitations of the Study

The main strength of this study is the comparison of a large cohort of patients affected by OLP with the general population. Furthermore, the multicentre nature of this study makes it representative of the entire Italian population.

Another strength of the study is that strict inclusion criteria were adopted; we excluded cases of oral carcinoma, as it do not fall within the aim of the study. Moreover, we also excluded all subtypes of H&N cancers, to avoid an overestimation of the cancer prevalence due to ‘field cancerization’. Therefore, we excluded possible confounders in both groups.

The results of this study must be carefully interpreted owing to its design, and cannot provide indications about the causality of the association between OLP and extraoral cancer. Thus, prospective studies must consider the temporal link between OLP diagnosis and extraoral cancer.

Another crucial aspect that warrants investigation is the impact of immunosuppressive therapy (both local and systemic) in patients with OLP on the increased risk of developing an extraoral cancer.

Another important limitation is the lack of comprehensive documentation and analysis of patients' health status and medical history during the interval between the initial cancer diagnosis and the onset of OLP, or vice versa. Given a long frame time, the onset of new comorbidities, treatments received, or lifestyle factors could play a role in the development of OLP.

Moreover, further studies should consider any risk factors shared by OLP and different types of neoplasia.

## 5 Conclusion

In conclusion, this is the first multicentre study to report a statistically significant association between OLP and the risk of extraoral cancer. Patients with OLP have a 1.79‐fold higher risk of extraoral cancer and suggest that erosive form and plaque‐like forms should be considered at higher risk. Potential pathogenic mechanisms involve a complex interplay between chronic inflammation, immune dysregulation and carcinogenesis. Therefore, while the association between OLP and cancer is of interest, its directionality remains unclear, underscoring the need for prospective studies to clarify causality and temporal dynamics.

## Author Contributions

**Gioele Gioco:** data curation. **Anna Schiavelli:** investigation. **Alessandra Majorana:** investigation. **Elena Bardellini:** investigation. **Silvio Abati:** investigation. **Andrea Sardella:** investigation. **Francesco Spadari:** investigation. **Matteo Pellegrini:** investigation. **Dorina Lauritano:** investigation. **Raffaele Borgia:** investigation. **Monica Pentenero:** investigation. **Samuele Sutera:** investigation. **Paolo Giacomo Arduino:** investigation. **Alessio Gambino:** investigation. **Roberto Broccoletti:** investigation. **Matteo Biasotto:** investigation. **Giulia Ottaviani:** investigation. **Lorenzo Azzi:** investigation. **Alessandro D'Aiuto:** investigation. **Davide Bartolomeo Gissi:** investigation. **Andrea Gabusi:** investigation. **Andrea Santarelli:** investigation. **Marco Mascitti:** investigation. **Paolo Vescovi:** investigation. **Gloria Bortolotti:** investigation. **Maddalena Manfredi:** investigation. **Marco Meleti:** investigation. **Grazia Zino:** investigation. **Umberto Romeo:** investigation. **Gianluca Tenore:** investigation. **Gianfranco Favia:** investigation. **Luisa Limongelli:** investigation. **Massimo Petruzzi:** investigation. **Francesca Dimilito:** investigation. **Lorenzo Lo Muzio:** investigation. **Maria Eleonora Bizzoca:** investigation. **Giuseppina Campisi:** investigation. **Vera Panzarella:** investigation. **Stefania Leuci:** investigation. **Rosario Serpico:** investigation. **Alberta Lucchese:** investigation. **Giuseppe Colella:** investigation. **Giacomo Oteri:** investigation. **Antonia Marcianò:** investigation. **Gaetano Isola:** investigation. **Alessandro Polizzi:** investigation. **Amerigo Giudice:** investigation. **Elena Calabria:** investigation. **Umberto Mariani:** investigation. **Daniela Adamo:** investigation. **Federica Canfora:** investigation. **Filippo Graziani:** investigation. **Marco Nisi:** investigation. **Michele Giuliani:** investigation. **Luisa Ritrovato:** investigation. **Federica De Micheli:** investigation. **Cosimo Rupe:** methodology, formal analysis, writing – review and editing. **Francesco Scilla:** visualization. **Romeo Patini:** writing – review and editing, visualization, validation. **Massimo Cordaro:** validation, supervision. **Michele Davide Mignogna:** investigation. **Carlo Lajolo:** conceptualization, methodology, data curation, validation, formal analysis, visualization, writing – original draft, writing – review and editing.

## Funding

The authors have nothing to report.

## Ethics Statement

The study was approved by the ethics committee of the promotion centre and satellite centres and was conducted in accordance with the principles of the Declaration of Helsinki (Prot. ID 4073; Clinicaltrial.gov NCT06449248).

## Conflicts of Interest

The authors declare no conflicts of interest.

## Supporting information


**Appendix S1:** Coordinating and satellite centres involved in this multicentre study.

## Data Availability

The data that support the findings of this study are available from the corresponding author upon reasonable request.
