# Supplementary material for: Association Between Oral Lichen Planus and Non‐Oral Cancers: A Multicentre Case–Control SIPMO Study
Source: Oral Dis. 2025 Nov 24;32(4):1054–67. doi: 10.1111/odi.70152 (PMC13248582; doi:10.1111/odi.70152)
Supplement: Supplementary file 1 — Appendix S1: Coordinating and satellite centres involved in this multicentre study. [file ODI-32-1054-s001.docx]

**Appendix 1. Coordinating and satellite centres involved in this multicentre study**

Coordinating center:

1 Fondazione Policlinico Universitario A. Gemelli IRCCS Università Cattolica del Sacro Cuore, Rome, Italy

Satellite centres

2 University of Trieste, Trieste, Italy

3 University of Bologna, Bologna, Italy

4 University of Brescia, Brescia, Italy

5 University of Ferrara, Ferrara, Italy

6 University of Campania Luigi Vanvitelli, Naples, Italy.

7 University of Palermo, Palermo, Italy

8 Cir-Dental School, University of Turin, Turin, Italy.

9 Oral Medicine and Oral Oncology Unit, University of Turin, Turin, Italy

10 School of Dentistry, University of Catanzaro, Viale Europa, Catanzaro, 88100, Italy

11 University of Rome "Sapienza", Rome, Italy.

12 University Center of Dentistry, University of Parma, Parma, Italy.

13 School of Dentistry, University of Catania, Catania, Italy.

14 University of Insubria, Varese, Italy

15 University of Foggia, Foggia, Italy

16 University of Bari “Aldo Moro”, Bari, Italy

17 University of Naples Federico II, Naples, Italy.

18 Fondazione IRCCS Ca' Granda Ospedale Maggiore Policlinico, University of Milan, Milan, Italy.

19 Marche Polytechnic University, Ancona, Italy.

20 University of Messina, Messina, Italy.

21 Dental School, IRCCS San Raffaele Hospital and University Vita-Salute San Raffaele, Milan, Italy
